# Prolonged Bioluminescence Monitoring in Mouse *Ex Vivo* Bone Culture Revealed Persistent Circadian Rhythms in Articular Cartilages and Growth Plates

**DOI:** 10.1371/journal.pone.0078306

**Published:** 2013-11-04

**Authors:** Naoki Okubo, Yoichi Minami, Hiroyoshi Fujiwara, Yasuhiro Umemura, Yoshiki Tsuchiya, Toshiharu Shirai, Ryo Oda, Hitoshi Inokawa, Toshikazu Kubo, Kazuhiro Yagita

**Affiliations:** 1 Department of Physiology and Systems Bioscience, Kyoto Prefectural University of Medicine, Kyoto, Japan; 2 Department of Orthopaedics, Graduate School of Medical Science, Kyoto Prefectural University of Medicine, Kyoto, Japan; 3 Department of Musculoskeletal Chronobiology, Graduate School of Medical Science, Kyoto Prefectural University of Medicine, Kyoto, Japan; 4 Department of Orthopaedic Surgery, Graduate School of Medical Science, Kanazawa University, Kanazawa, Japan; 5 Precursory Research for Embryonic Science and Technology (PREST), Japan Science and Technology Agency, Saitama, Japan; University of Insubria, Italy

## Abstract

The bone is a metabolically active organ which undergoes repeated remodeling cycles of bone resorption and formation. In this study, we revealed a robust and extremely long-lasting circadian rhythm in *ex vivo* culture maintained for over six months from the femoral bone of a PERIOD2^Luciferase^ mouse. Furthermore, we also identified robust circadian clocks in flat bones. High- or low-magnification real-time bioluminescence microscopic imaging revealed that the robust circadian rhythms emanated from the articular cartilage and the epiphyseal cartilage within the growth plate of juvenile animals. Stimulation by forskolin or dexamethasone treatment caused type 0 phase resetting, indicating canonical entraining properties of the bone clock. Together, our findings from long-term *ex vivo* culture revealed that “tissue-autonomous” circadian rhythm in the articular cartilage and the growth plate of femoral bone functions for several months even in an organ culture condition, and provided a useful *in vitro* assay system investigating the role of the biological clock in bone formation or development.

## Introduction

The bone is a metabolically active tissue which undergoes repeated cycles of bone remodeling including bone resorption and formation. These two opposing processes are tightly controlled to maintain homeostasis, and bone metabolic disturbances can cause severe diseases. For example, excess bone resorption leads to osteoporosis and increases bone fracture risk [1], [2]. Several biochemical markers have been identified for the remodeling process. Bone alkaline phosphatese, osteocalcin, and procollagen peptides are known as bone formation markers, whereas tartrate-resistant acid phosphate, collagen brakedown products, and cross-linked telopeptides (NTX and CTX), deoxypyridirinolin (Dpd) as bone resorption markers [3]. Notably, levels of certain markers, such as osteocalcin, NTX, CTX and Dpd, are reported to show diurnal fluctuation in plasma or urine [4]–[6]. For example, the parathyroid hormone (PTH) promotes bone resorption, and its serum level was found to exhibit a diurnal variation with night-time peaks [7]. El-Hajj Fuleihan *et al*. found that the plasma PTH rhythm persisted under “constant routine” conditions where subjects were deprived of any exogenous time information, indicating that the rhythm is driven by the intrinsic circadian clock [8].

The circadian clock is an endogenous oscillator which generates approximately 24-hour biological cycles. At the molecular level, the circadian clock is composed of a set of clock genes forming cell-autonomous transcription/translation feedback loops; the molecular oscillators in turn drive the expression of output genes governing a variety of clock-controlled physiological processes [9], [10]. Specifically, two transcription factors, BMAL1 and CLOCK, heterodimerize and transactivate core clock genes such as *Period* genes (*Per1* and *Per2*), *Cryptochome1* (*Cry1*), and *Rev-Erb* genes (*Rev-Erbα* and *Rev-Erbβ*) [11]–[13]. Expressions of these genes (*Bmal1*, *Per1*, *Per2*, *Cry1*, *Cry2*, *RevErbα* and *RevErbβ*) show clear circadian rhythms with distinct peak times [11]–[13]. Importantly, clock gene oscillation is not only observed in the pacemaker neurons of the hypothalamic suprachiasmatic nucleus (SCN) but also in almost all peripheral tissues [14], [15] and cultured cells such as Rat-1 rat fibroblast [16], NIH-3T3 mouse fibroblast [17], and U2OS human osteosarcoma cell lines [18]. Importantly, the molecular clock machinery in cultured cells is essentially identical to that of the SCN [19].

The molecular clock mechanism in bone physiology remains largely unclear. Previously, Fu *et al*. showed that mice lacking both *Per1* and *Per2* or both *Cry1* and *Cry2* displayed an exaggerated bone mass and increased numbers of osteoblasts [20]. In comparison, Maronde *et al*. reported that mice lacking a functional *Per2* gene led to increased bone formation, and a disruption of the *Cry2* gene did no change osteoblastic markers but caused attenuation of osteoclast markers [21]. Although these results suggest mechanistic cross-talk between the circadian clock and bone metabolism, the bone circadian clock at the organ level remains to be characterized.

Real-time bioluminescence monitoring of organotypic cultures can reveal intrinsic oscillation devoid of systemic time cues. PERIOD2^Luciferase^ knock-in (PER2^Luc^) mice carry a firefly *Luc* open-reading frame inserted immediately in front of the stop codon of the endogenous *Per2* gene [15]. Yoo *et al*. observed that PER2::Luc activity showed clear circadian rhythms in cultured SCN and other peripheral tissues. Moreover, they also found that ablation of the SCN augmented circadian phase variation in peripheral tissues compared to that from animals with intact clocks, underscoring an important role of the SCN in the synchrony of peripheral clocks [15].

Peripheral tissues such as the liver, lung, pituitary, and kidney all exhibit robust circadian rhythms in culture [15], [22]. Therefore, it is conceivable that bones, with diurnal metabolic oscillation, also have a circadian clock. In this study, we performed long-term real-time monitoring of *ex vivo* bone culture using PER2^Luc^ mice to directly demonstrate a tissue-autonomous bone circadian clock. Furthermore, we evaluated the phase-resetting effects of forskolin and dexamethasone (DEX) on the bone PER2::Luc rhythms.

## Materials and Methods

### Ethics Statement

Animal experiments were performed with approval from the Experimental Animals Committee, Kyoto Prefectural University of Medicine (No. M22-270).

### Animals and bone sampling

The PER2^Luc^ K.I. mice were originally developed by Dr. Joseph Takahashi's group [15] and maintained in our facility. Both heterozygote and homozygote mice were used. Mice were maintained under 12 hour light (7:00–19:00) – 12 hour dark (19:00–7:00) conditions, and food and water were available *ad libitum*.

For tissue sampling, mice (3–9 weeks old) were deeply anesthetized with sodium pentobarbital and sacrificed. After sterilization with 70% ethanol, bones were collected and stored in ice-cold PBS. Muscle tissues or tendons were carefully removed by scissors. The femoral bone was divided into halves and cultured as a distal or proximal femoral end. Calvarium was trimmed to remove soft tissues.

### Bone organ culture and real-time bioluminescence monitoring

Bone organ culture was performed as previously reported [15]. In brief, collected bones were placed in a 35 mm cell culture dish containing 1.2 ml of the culture medium. The culture medium contained the phenol red-free D-MEM (Nacalai tesque), 1x Glutamax (Life Technologies), 1x B-27 supplement (Life Technologies), 10 mM HEPES, 200 µM beetle luciferin (Promega), 100 units/ml Penicillin and 100 µg/ml streptomycin (Nacalai tesque). After being sealed with parafilm (Pechiney Plastic Packaging), the culture dishes were placed in the photomultitipler tube (PMT) based on real-time bioluminescence monitoring equipment [23]and maintained at 35°C.

### Microscopic observation

Microscopic observation was performed using a high-sensitivity charge-coupled device (CCD) camera-based microscopic image analyzer (Olympus). We used femoral samples from which we had observed the circadian bioluminescence rhythm using PMT based real-time bioluminescence monitoring equipment. After several days in the culture (6 days for Movie S1 (16.5 weeks old mouse) or 4 days for Movie S2 (2.4 weeks old mouse)), the femoral sample was set to the CCD camera-based microscopic image analyzer. For synchronization purposes, the bone was treated with forscolin (10 µM for one hour), and then placed with a fresh culture medium. For time series analysis, bone images were obtained every hour. An image analysis was performed using the Aqua Cosmos software (Hamamatsu Photonics) according to the manufactures' instruction.

### Chemical stimulation

Both forskolin (Sigma) and dexamethasone (DEX) (Sigma) were dissolved in ethanol (EtOH) and used at 10 µM and 100 nM final concentrations, respectively. Chemical stimulations were carried out as previously reported [24]. In brief, bone samples were synchronized with forskolin (10 µM) for 1 hour and thereafter released into a fresh culture medium. Chemicals were added after one full circadian cycle (peak to peak) of PER2::Luc activity.

### Data Analysis

For calculating the circadian period and circadian phase of several types of bones, data were detrended by a 24-hour moving average and analyzed with an RAP program [25]. For detecting the phase shift, data were detrended by 24-hour moving average and the damping effect was corrected by using a 24-hour standard deviation [26]. The circadian period was calculated as the peak-to-peak interval, and the difference in the periods between pre- and post-stimulation was calculated.

## Results

### Prolonged circadian rhythms in bone culture

**Figure 1:**
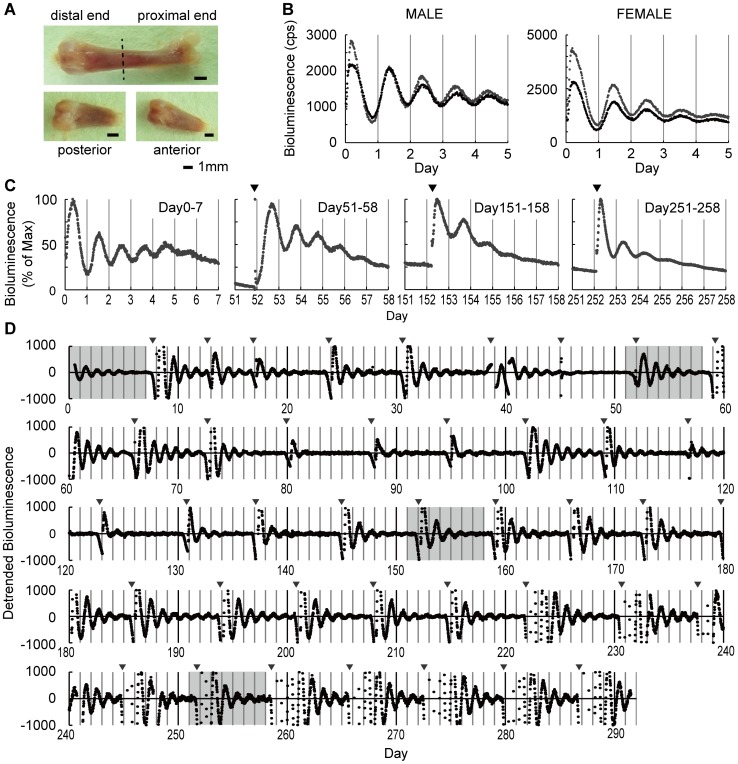
Persistent cell-autonomous oscillators in the bone. (**A**) Mouse femurs harvested from PER2^Luc^ mice. Distal femoral ends were used. (**B**) Two representative (black and grey dots) bioluminescence circadian rhythm traces. (**C–D**) Medium change restored circadian oscillation after 280 days in *ex vivo* culture. Four representative periods are shown where data are expressed as a percent of the max value in 7 days (**C**), and the entire 292 days of *ex vivo* culture data are shown in **D**. Data were detrended by a 24-hour moving average and the grey area is equivalent to data shown in **C**. Arrowheads indicate medium change.

To demonstrate the presence of an autonomous circadian clock in the bone, we performed a real-time luminescence recording using PER2^Luc^ mice. The distal half of the femoral bone was harvested from heterozygote PER2^Luc^ mice and cultured in 35 mm cell culture dishes, and bioluminescence was measured every 20 minutes. Bioluminescence from the femoral bone showed a clear circadian rhythm from both female and male mice (**Figure 1B**). The amplitude of the circadian bioluminescence rhythm gradually dampened, similar to that observed in other peripheral tissues [14]. Importantly however, a medium change restored bone circadian oscillation even after 280 days, suggesting a *bona fide* tissue-autonomous circadian clock in the bone (**Figure 1C**).

**Figure 2:**
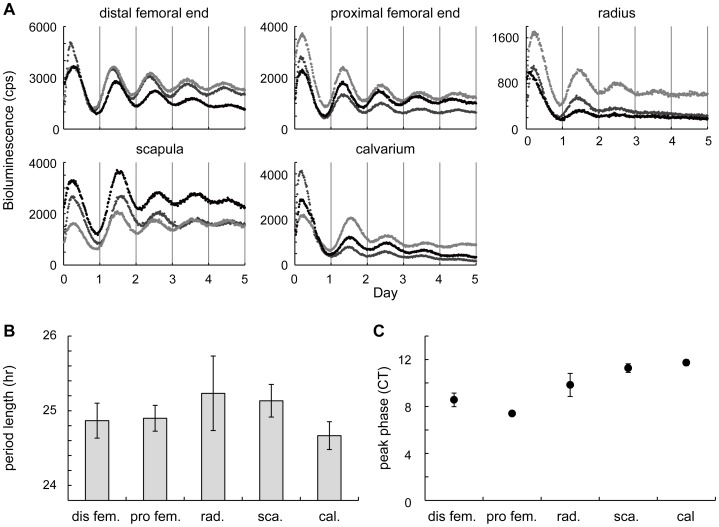
Circadian clocks in different bone tissues. (**A**) Bioluminescence circadian rhythms obtained from the distal femoral end, proximal femoral end, radius, scapula, and calvarium. Color dots (light gray, gray and black) indicate traces from three independent samples. (**B–C**) The quantitative analysis of circadian bioluminescence rhythms. The period length (**B**) and bioluminescence peak phase (**C**) are shown (mean ± SEM, n = 3). For peak phase calculation, CT0 was set as a start time of the measurement. CT: Circadian time; dis fem: distal femoral end; pro fem: proximal femoral end; rad: radius; sca: scapula; cal: calvarium.

In addition to the femoral bone, other bone tissues also showed autonomous reporter oscillation. In the culture, circadian bioluminescence rhythms from long bones (proximal femoral ends and radiuses) and flat bones (calvariae and scapulae) showed similar period lengths (**Figure 2A–B**). Peak phases were slightly different between bones: peak times of both distal and proximal femoral ends were similar (CT 8.4±0.34 and 7.4±0.12 respectively, mean ± S.E.M) whereas those of scapulae and calvariae were delayed by several hours (CT11.3±0.37 and 11.7±0.26, respectively). Radiuses showed an intermediate peak time (CT9.8±0.98) (**Figure 2C**).

### Localization of bioluminescence circadian rhythms in PER2^Luc^ mouse femur

**Figure 3:**
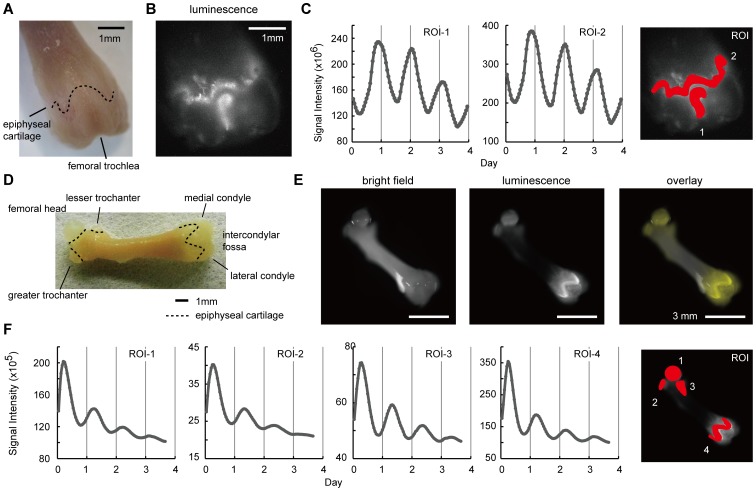
Microscopic observation of the femur. (**A**) The overview of the distal half of the femur. The dotted line indicates epiphyseal cartilage. The mirror-reversed image was used for comparison to the bioluminescence image. A sample was obtained from a 16.5-week old mouse and cultured for 6 days before observation. (**B**) The bioluminescence image obtained by a microscope-based high sensitivity CCD camera system. (**C**) A time series analysis of the epiphyseal cartilage (**ROI-1**) and femoral trochlea (**ROI-2**). The right panel shows set ROIs. (**D**) A schematic view of the femur. The dotted line indicates epiphyseal cartilage. A sample was obtained from a 2.4-week old mouse and cultured for 4 days before observation. (**E**) A representative microscopic view of the femur. A bright field (**left**), bioluminescence (**middle**), and overlaid image (**right**) are displayed. For the overlaid image, the bioluminescence signal is shown in yellow. (**F**) A time series analysis of the signal intensity for 88-hours. The right panel shows set ROIs.

To further delineate the spatial distribution of bioluminescence in femoral bone tissues, we used a microscope-based high-sensitivity CCD camera imaging system (Olympus) (**Figure 3A–C**). High-magnification bioluminescence microscopy was performed to focus on the distal epiphysis where both articular cartilage and mineralized bone were monitored. A strong signal existed in the epiphyseal cartilage of the growth plate and articular cartilage where chondrocytes covered the bone surface. Time series analysis of signal intensity revealed that both epiphyseal cartilage (ROI-1) and articular cartilage in femoral trochlea (ROI-2) showed clear circadian rhythms for 4 days (**Figure 3B–C** **, Movie S1**).

Next we performed low-magnification real-time bioluminescence microscopy to observe circadian rhythms in the whole femoral bone. Interestingly, PER2::Luc bioluminescence strongly oscillated in the epiphyseal cartilage of growth plates located in both distal and proximal ends of the femoral bone (**Figure 3D–F**). Time series analysis revealed that the femoral head (ROI-1), greater trochanter (ROI-2), lesser trochanter (ROI-3) and epiphyseal cartilage of the distal femoral end (ROI-4) showed a circadian rhythm for 4 days (**Figure 3F** **, Movie S2**). Although expression of certain clock genes has been shown in growth plates [27], [28], our results constitute the first observation of autonomous circadian rhythms in growth plates. Our observations also suggest that local circadian clocks in the epiphyseal cartilage may affect bone growth in juvenile animals. In addition, the microscopic observation of femur bioluminescence revealed strong signals in the cartilage. The growth plate of long bones consists of a glacial cartilage called epiphyseal cartilage, and endochondral osscification in the epiphyseal cartilage leads to bone growth [29]. In a recent report, clock genes and the type II collagen gene have been shown to display rhythmic expression in the growth plate of femur *in vivo* [27]. These observations overall suggest a cross-talk between the circadian clock and bone growth, although further studies are needed to delineate the detailed mechanism.

### Bone clock entrainment by forskolin and dexamethasone

Exogenous time cues (*e.g.* light) can reset internal clocks. We next studied whether the bone clock can be reset by chemical stimulation. Forskolin and DEX were tested because both chemicals are known to reset circadian clocks in fibroblast [26], [30], [31] and cultured organs like the lung, liver or cardiac atria [24], [32].

**Figure 4:**
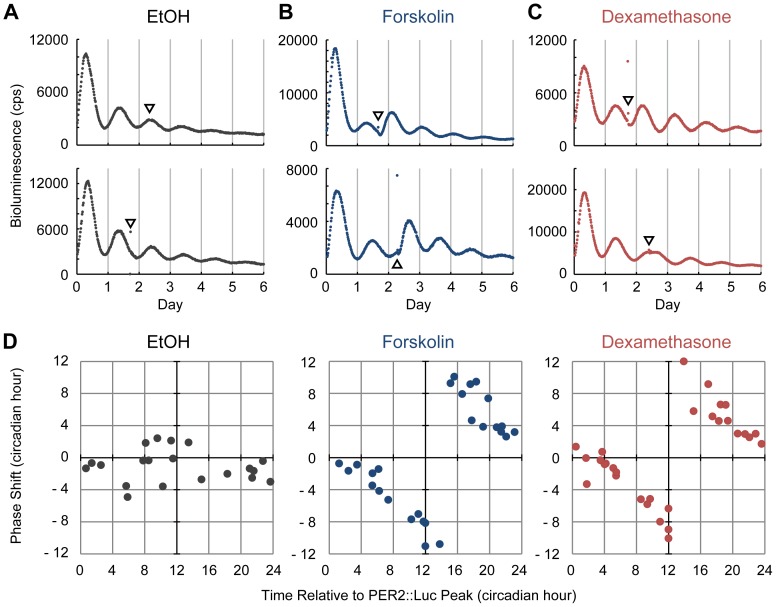
Phase resetting of bone circadian clocks by chemicals. (**A–C**) Effects of forskolin and dexamethasone (DEX) on circadian clocks. Representative data showing the phase advancement (**upper panels**) or phase delay of the bone circadian clock (**lower panels**). The arrowhead indicates chemical administration. From left to right, the vehicle (ethanol; EtOH) (**A**), DEX (**B**), or forskolin (**C**). (**D**) Phase response curves for the vehicle (left), forskolin (middle) or DEX (right). Results of 20 (vehicle), 27 (forskolin), and 30 (Dex) experiments were plotted, respectively. The horizontal axis represents time relative to PER2::Luc peak time (CT12) and the vertical axis represents phase shift. Data are represented in circadian hours (1 circadian hour  =  period length (hour) /24).

Administration of vehicle (EtOH) slightly affected the circadian rhythm (**Figure 4A**); in comparison, we observed significant phase shifts in response to forskolin and DEX (**Figure 4B–C**). When forskolin was administered following the PER2::Luc activity peak, the circadian phase was advanced; in other words, the next peak occurred earlier than expected (**Figure 4B** **upper panel**). In contrast, when forskolin was administered before the PER2::Luc activity peak, the PER2::Luc phase was delayed (**Figure 4B** **lower panel**). Similar to forskolin, administration of DEX before or after the PER2::Luc peak resulted in phase delay or advancement, respectively (**Figure 4C**). The phase response curve (PRC) of both forskolin and DEX showed type 0 PRCs without an irresponsive period for the stimuli throughout the circadian cycle (**Figure 4D**). Although the PRCs are similar in shape, the “breakpoint” in the DEX-induced PRC is slightly earlier than that of the forskolin-induced PRC.

## Discussion

In this study, we showed that PER2::Luc activity in bones displayed circadian periodicity (24.3 to 26-hr period length in different bones) in the culture, and this oscillation is cell-autonomous, as the bioluminescence rhythm was observed for more than nine months. Although other groups reported circadian rhythms of clock gene expression in the femur [27], [33], vertebrate [33], calvarium [34], and rib growth plate [28] using the quantitative PCR method following time-course sampling, our data showing a long-lasting circadian rhythm observed from *ex vivo* culture of bones provided definitive evidence that bone tissues, including the growth plate and the articular cartilage, express self-sustained and cell-autonomous circadian rhythms.

In this study, we have also shown that phase of the bone clock was strongly altered by forskolin which functions to increase intracellular cAMP levels through adenylate cyclase activation. Therefore, it is possible that *in vivo* circadian phase can be reset by endogenous substance(s) via the cAMP pathway in the bone. The hormone PTH increases intracellular cAMP levels via the PTH/PTHrP receptor, consequently inducing *Per1* and *Per2* expression through the cAMP–PKA–CREB pathway [27]. In addition, sympathetic signaling stimulated by leptin has been shown to regulate bone remodeling in part through a β-adrenergic receptor (β-AR) [35]. The β-AR agonist isoproterenol also enhances intracellular cAMP levels and up-regulates *Per1/2* and *Bmal1* expression in primary mouse osteoblasts [20]. In accordance, isoproterenol has been reported to stimulate the circadian rhythmic expression of *Per1/2/3* and *Bmal1* in human SaM-1 osteoblastic cells [36].

DEX, synthetic glucocorticoids (GCs), was found to reset the bone circadian rhythm. GCs are secreted from the adrenal gland in a circadian manner [9]. Although the SCN is not reset by GCs due to a lack of glucocorticoid receptor (GR) expression, circadian clocks in peripheral organs such as the liver, kidney, and heart are highly responsive to GCs [31]. GCs are considered as internal time-cues which relay timing information within the body and synchronize the peripheral clocks, including bones, as shown here. At the molecular level, GCs bind to GR and regulate target gene expression via glucocorticoid response elements. Previous studies have identified *Per1*, *Per2*, and *E4bp4* as direct targets of GRs in mice [37].

Forskolin and DEX modulate the bone clock in a time-dependent manner. Comparison of forskolin-induced PRC and DEX-induced PRC showed a slightly advanced “break point” in the DEX-induced PRC, most likely due to distinct signaling cascades targeted by these chemicals [26], [36].

GCs are known to have inhibitory effect on the growth plate [38] where we found high PER2::Luc activity. Thus the systemic hormonal factors including GC may contribute the regulation of bone metabolism and/or growth to some extent. On the other hands, as shown in this study, we demonstrated that the extremely robust local circadian clock oscillation in the growth plate in the femur explants cultured for several months. Therefore, the correlation between local circadian clock and systemic circadian rhythms such as hormonal rhythms should be unveiled to understand the regulating mechanism of bone growth in further studies.

## Conclusion

We demonstrated here that both long and flat bones harbor cell-autonomous molecular clocks driving circadian rhythms throughout the nine-month experimental duration. We also showed that bone clocks could be reset by both forskolin and the synthetic glucocorticoid DEX in a time-specific manner. These results suggest that hormones, including glucocorticoids, most likely function as internal time-cues for the bone clock. In addition, we also showed that the epiphyseal cartilage in the growth plate expressed a robust tissue-autonomous circadian rhythm.

## Supporting Information

Movie S1 Related to **Figure 3**. High-magnification bioluminescence microscopy of the distal epiphysis. A clear circadian rhythm of the bioluminescence was observed in medial condyle and femoral trochlea, as shown in **Figure 3C**. The time scale shown on the lower right corner indicats “hours: minutes: seconds”.
(AVI)

Movie S2 Related to **Figure 3**. The real-time bioluminescence imaging of the femur using low-magnification bioluminescence microscopy. A clear circadian rhythm of the bioluminescence was observed in the epiphyseal cartilage, as shown in **Figure 3F**. The time scale shown on the lower left area indicats “hours: minutes: seconds”.
(AVI)
